# Non-invasive Quantitative Assessment of Layer-Specific Degeneration in the Lumbar Multifidus in Elite Male High School Soft Tennis Players Using Ultrasound Echo Intensity

**DOI:** 10.7759/cureus.92283

**Published:** 2025-09-14

**Authors:** Toru Tanabe, Tomonari Sugano, Takumi Watabu, Katsunori Mizuno

**Affiliations:** 1 Department of Physical Therapy Rehabilitation, Fukui General Clinic, Fukui, JPN; 2 Department of Rehabilitation, Faculty of Health Science, Fukui Health Science University, Fukui, JPN; 3 Department of Orthopedic Surgery, Fukui General Hospital, Fukui, JPN

**Keywords:** adolescent athletes, echo intensity, lumbar multifidus, muscle degeneration, soft tennis, ultrasound imaging

## Abstract

Introduction

Soft tennis involves repetitive, high-intensity trunk rotation during strokes and serves, with high popularity among Japanese high school boys. The sport’s biomechanical demands place cumulative stress on the lumbar spine, and a high prevalence of low back pain (LBP) has been reported, particularly in elite-level athletes. Previous studies identified factors such as hip rotation range of motion (ROM), trunk strength, and training load as LBP-related in rotational sports. However, foundational insights into the structural characteristics of the lumbar region in this population remain limited, hindering the identification of anatomical risk factors for LBP.

The lumbar multifidus (MF) is a key deep stabilizing muscle contributing to dynamic spinal control. It consists of superficial and deep layers, with the deep layer playing a primary role in segmental stability. Due to this role, the deep MF may be more susceptible to degeneration, such as fatty infiltration, under repeated sport-specific load.

Objective

This study aimed to non-invasively assess structural differences between superficial and deep layers of the lumbar MF in elite male high school soft tennis players, using ultrasound-based echo intensity (EI) as a quantitative index of tissue composition.

Methods

A cross-sectional study was conducted on 55 elite male high school soft tennis players. B-mode ultrasound (Noblus, Hitachi Aloka, Tokyo, Japan) images were acquired at the L4 level in a prone position, using standardized settings (depth 6.0-8.0 cm, frequency 10 MHz, gain 60 dB, and dynamic range 70 dB).

Using ImageJ (National Institutes of Health, Bethesda, MD, US), the MF was bisected from the superficial border to the lamina to define superficial and deep regions of interest (ROIs), and each ROI was manually traced. EI was defined as the mean grayscale value (0-255) within each ROI. Two images per side (dominant and non-dominant) were averaged. Two-way repeated-measures analysis of variance (ANOVA) tested the effects of layer and side, with significance at p < 0.05.

Results

A significant main effect of layer was found (F(1, 54) = 120.64, p < 0.001, partial η² = 0.691), with the deep layer showing higher EI than the superficial layer. No significant main effect of side or interaction was observed (p > 0.05). The mean superficial vs. deep EI was 89.84 vs. 115.69 on the non-dominant side, and 89.59 vs. 113.68 on the dominant side.

Conclusion

This study is the first to demonstrate consistently higher EI in the deep MF compared to the superficial layer, regardless of side, in elite male high school soft tennis players. These findings suggest that the deep MF, which is vital for spinal stability, may undergo degenerative changes due to repeated loading. Such structural imbalance may compromise segmental spinal control and increase future LBP risk. This study underscores the value of layer-specific EI as a potential biomarker and highlights the need for longitudinal research to explore its predictive utility for LBP in rotational athletes.

## Introduction

Soft tennis is a racket sport widely practiced in Asia and increasingly recognized internationally [1-3]. The court dimensions are the same as those of lawn tennis, but the use of a rubber-based soft ball and the dominance of doubles play create a distinctive playing style [1,4].

In Japan, soft tennis is highly popular among high school boys, and some elite athletes continue at the professional or corporate level [4]. However, the prevalence of low back pain (LBP) among male high school players is notably high. LBP accounts for 14% of all sports-related injuries among female athletes but rises to 28% in male athletes [5]. This disparity may be explained by the greater training load, higher match intensity, and larger physical demands typically placed on male athletes, which increase cumulative mechanical stress on the lumbar spine. In other sports, the prevalence among high school boys ranges from 24% to 36%, confirming LBP as one of the most frequent and burdensome injuries in this population [6]. LBP during adolescence not only limits participation and performance but also impairs long-term athletic development [6]. This risk is particularly evident in sports such as soft tennis, which involve frequent trunk rotations and impose cumulative mechanical stress on the lumbar spine [7-9].

Previous studies have identified several contributing factors to LBP in rotational sports, such as limited hip internal rotation, reduced trunk muscle strength, prior lumbar injury, and high training volume [7-11]. However, these investigations have mainly addressed functional or external risk factors, while relatively little attention has been paid to structural characteristics of the lumbar region itself.

The lumbar multifidus (MF) plays a central role in maintaining spinal stability during rotational sporting activities [10]. It consists of superficial and deep layers with distinct roles: the superficial layer spans multiple vertebral segments to support gross trunk movements, while the deep layer attaches near the vertebral arches and facet joints to provide fine intersegmental control [12]. In chronic LBP patients, impairments of the MF-including atrophy, fatty infiltration, and delayed activation-have been reported [13,14]. Yet, most studies have treated the MF as a homogeneous muscle, with limited exploration of differences between the superficial and deep layers.

Histological studies have shown that the deep MF contains a higher proportion of type I fibers, suited for sustained low-load contractions [15]. However, these fibers may adapt poorly to repeated, high-load, instantaneous stress, rendering the deep layer more vulnerable to degeneration despite its stabilizing function. Such degeneration, defined here in a restricted sense as structural alterations including fatty infiltration and fibrosis [16], and considered to result primarily from repetitive overuse rather than advanced pathological changes, may compromise spinal stability and increase LBP risk.

Ultrasound-derived echo intensity (EI), validated as a non-invasive marker of muscle composition and shown to correlate with magnetic resonance imaging (MRI) and histological findings [17,18], offers a practical means of assessing these alterations in adolescent athletes. In the adolescent population, even subtle degenerative tendencies may be clinically meaningful because the musculoskeletal system is still developing. Early structural alterations of the deep MF could represent an initial sign of spinal vulnerability, predisposing young athletes to recurrent or chronic LBP later in their careers and underscoring the importance of timely preventive strategies.

To date, no study has examined whether the deep MF already exhibits relative degeneration compared with the superficial layer in adolescent athletes. Establishing this baseline is essential before investigating its causal role in LBP. This study aimed to determine whether elite male high school soft tennis players show a degenerative tendency in the deep lumbar MF compared with the superficial layer, using ultrasound-derived EI as a non-invasive index. This preliminary investigation provides baseline evidence toward identifying structural risk factors for LBP in this population.

## Materials and methods

Study design and participants

This study employed a cross-sectional observational design and recruited 55 male high school soft tennis players from seven teams that had qualified for national-level tournaments. The inclusion criteria were as follows: (1) no history of surgical procedures involving the spine or extremities, (2) no prior diagnosis of organic lumbar pathologies such as lumbar disc herniation or spondylolysis, and (3) absence of neurological symptoms in the lower extremities (e.g., numbness, radiating pain, or muscle weakness suggestive of radiculopathy).

The study protocol was approved by the Ethics Committee of Nittazuka Medical and Welfare Center, and all procedures adhered to the principles of the Declaration of Helsinki. For participants under the age of 20, written informed consent was obtained from both the individual and their legal guardian.

In addition, baseline demographic and clinical information was recorded for each participant, including age, height, body weight, years of competitive experience, current medical conditions, and past medical history. The dominant side was defined as the upper limb used to hold the racket during a forehand stroke, and the contralateral side was designated as the non-dominant side.

Ultrasound imaging protocol

Ultrasound imaging of the MF was performed at the level of the fourth lumbar vertebra (L4) using a transverse short-axis view in B-mode. A high-resolution ultrasound diagnostic device (Noblus, Hitachi Aloka, Tokyo, Japan) was employed, and all measurements were acquired under standardized imaging parameters previously demonstrated to provide high measurement reliability. Participants were placed in a relaxed prone position on the examination table, with a cylindrical cushion positioned beneath the pelvis to reduce excessive lumbar lordosis and help maintain a neutral spinal alignment. Both arms were positioned comfortably alongside the trunk to minimize muscular tension during imaging.

**Figure 1:**
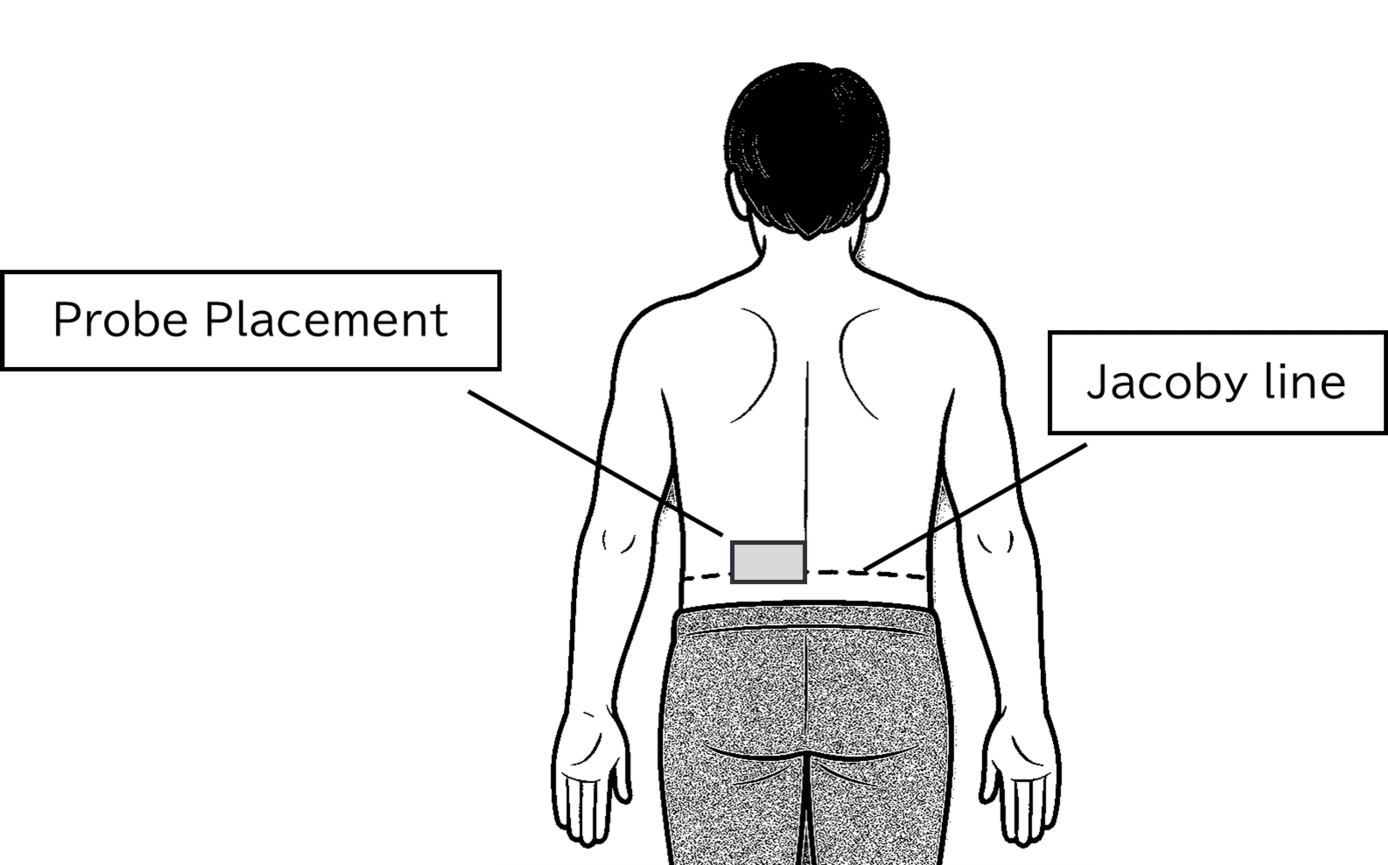
Schematic illustration of ultrasound probe placement based on the Jacoby line. The probe was positioned horizontally at the L4 level, along the Jacoby line, on each side of the lumbar spine. All imaging followed standardized procedures to ensure reproducibility across participants. Figure created by the authors.

**Figure 2:**
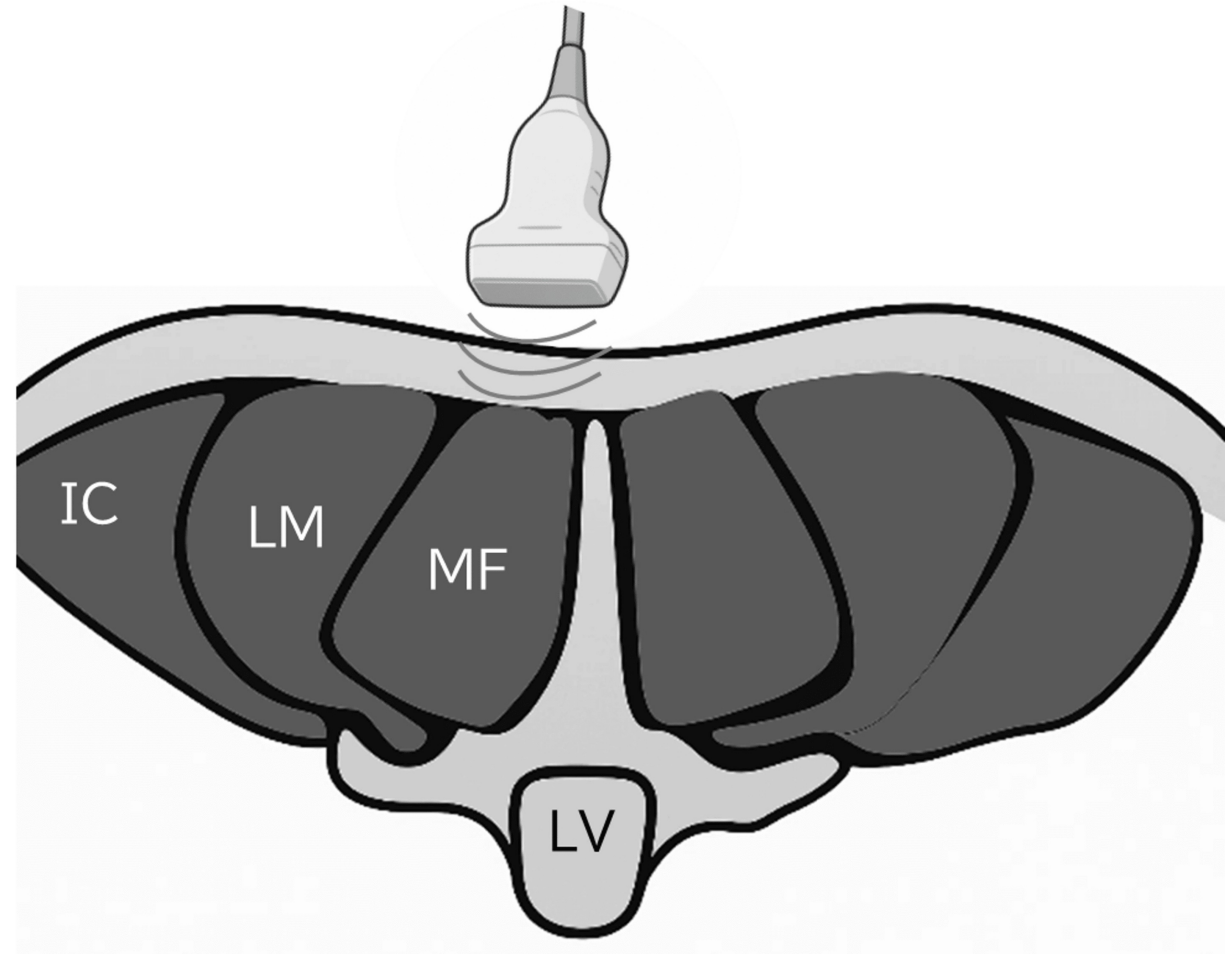
Schematic illustration of the cross-sectional anatomy of the lumbar multifidus muscle at the L4 level, including the placement of the ultrasound probe. The image illustrates the relative positions of the multifidus, longissimus, and iliocostalis muscles, as well as the underlying lumbar vertebra. All imaging procedures were standardized to enhance reproducibility. Cross-sectional anatomy illustrated based on reference [19]. Figure created by the authors. IC: iliocostalis muscle; LM: longissimus muscle; MF: multifidus muscle; LV: lumbar vertebra

To locate the L4 level, the Jacoby line-defined by connecting the superior borders of the bilateral iliac crests-was used as a surface anatomical reference (Figure 1). The L4 spinous process was palpated and marked, after which a linear-array transducer (8-12 MHz, 38 mm width) was placed vertically just lateral to the spinous process (Figure 2). The imaging level was reconfirmed through identification of osseous landmarks, including the vertebral arch and facet joints.

Ultrasound settings were standardized across all participants. The transducer was set to a frequency of 10 MHz, and the image depth was adjusted within a range of 4.0 to 6.0 cm, depending on individual anatomical variation, to ensure complete visualization of the MF from the superficial fascia to the vertebral arch. The gain was maintained at 50 dB, and the dynamic range was set to 70 dB. The focal zone was aligned with the mid-depth of the MF, corresponding to the center of the muscle belly, to achieve balanced image clarity across both the superficial and deep muscle layers. Speckle reduction was set to medium, harmonic imaging was turned off, and the time gain compensation (TGC) curve was centered and evenly spaced. Image brightness and contrast were not adjusted individually between participants; the same preset was applied for all acquisitions to ensure consistency.

For each side (dominant and non-dominant), two static images were captured, with the probe repositioned between acquisitions to minimize potential bias from transducer placement. All ultrasound examinations were conducted by a single licensed physical therapist with over five years of experience in musculoskeletal ultrasonography. An assistant was present throughout the procedure to assist with maintaining proper probe orientation and participant positioning, ensuring consistent image quality across all subjects.

Image analysis and calculation of EI

**Figure 3:**
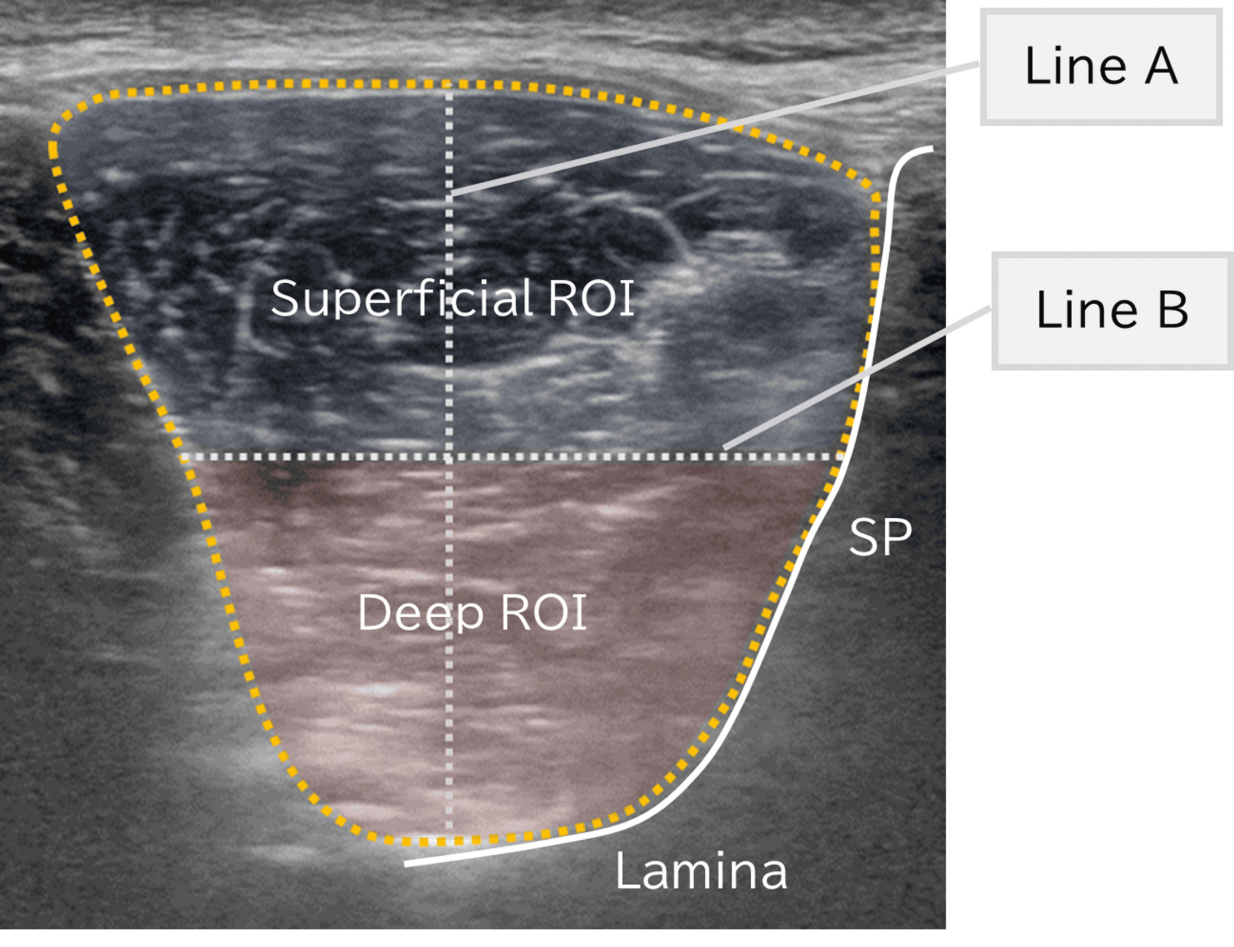
Schematic representation of superficial and deep regions of interest (ROIs) in the lumbar multifidus muscle on ultrasound image at the L4 level. Line A connects the most superficial border of the multifidus to the lamina. Line B is drawn perpendicular to the midpoint of Line A, dividing the muscle into superficial and deep ROIs. The region enclosed by the yellow dotted line represents the entire multifidus cross-section. ROI definitions were performed using standardized procedures to ensure reproducibility. SP: spinous process

All acquired ultrasound images were saved in DICOM format and analyzed using ImageJ software (version 2.0, National Institutes of Health, Bethesda, MD, US). In each image, a straight reference line was drawn from the most superficial point of the MF to the base of the L4 vertebral arch, taking into account relevant anatomical landmarks. This line was then bisected to define the superficial and deep layers with equal vertical depth (Figure 3). The superficial layer was defined as the external portion adjacent to the thoracolumbar fascia, while the deep layer was defined as the internal portion adjacent to the vertebral arch and facet joints.

This division method was based on the layer-specific fiber architecture of the MF: the superficial fibers originate from the spinous processes and span multiple vertebral levels, whereas the deep fibers insert onto the laminae and span only one or two vertebral segments [12]. Regions of interest (ROIs) for both layers were manually traced using the polygon selection tool in ImageJ. Non-muscle structures such as fascia, subcutaneous fat, and bone were carefully excluded.

In this study, EI was defined as the mean grayscale value of all pixels within the ROI, ranging from 0 (black) to 255 (white). All ROIs were delineated by the same examiner to ensure consistency. For each participant, the EI values were averaged across two images per side (dominant and non-dominant), for each layer, to obtain representative values.

This method is widely recognized for its high reliability as an indicator of intramuscular fatty infiltration and fibrotic degeneration. Furthermore, EI has been shown to correlate strongly with fat fraction measurements obtained via MRI as well as histological findings from muscle biopsies [17,18].

To evaluate measurement reliability, a test-retest analysis was conducted using ultrasound data from 10 subjects who did not participate in the main study. A seven-day interval was observed between sessions. All imaging and ROI selections were performed by the same examiner using identical procedures as in the main study. Intraclass correlation coefficients (ICCs) were calculated to assess intra-rater reliability. The results demonstrated excellent reproducibility: superficial layer: ICC = 0.95 (95% confidence interval (CI): 0.91-0.98); deep layer: ICC = 0.97 (95% CI: 0.94-0.99).

Statistical analysis

All statistical analyses were performed using Excel Statistics 2023 (Social Survey Research Information Co., Ltd., Tokyo, Japan). EI values are reported as mean ± standard deviation (SD).

To assess the main and interaction effects of the within-subject factors, a two-way repeated-measures analysis of variance (two-way RM-ANOVA) was conducted with "layer" (superficial vs. deep) and "side" (dominant vs. non-dominant) as the two within-subject factors. Prior to performing ANOVA, the Shapiro-Wilk test was used to verify the normality of the data distribution. When appropriate, Mauchly’s test of sphericity was performed to assess the assumption of sphericity. If this assumption was violated, the Greenhouse-Geisser correction was applied. Effect sizes for the ANOVA were reported using partial eta squared (η²) and interpreted according to conventional benchmarks: small (η² = 0.01), medium (η² = 0.06), and large (η² = 0.14) [20]. In cases where a significant interaction effect was observed, post hoc analyses of simple main effects were conducted using paired t-tests. The Bonferroni correction was applied to adjust for the inflation of type I error due to multiple comparisons. Cohen’s d was also calculated to evaluate the magnitude of differences in pairwise comparisons, with interpretations based on the following thresholds: small (d = 0.2), medium (d = 0.5), and large (d = 0.8) [20].

The required sample size was determined a priori using G*Power version 3.1 (Heinrich Heine University, Düsseldorf, Germany), based on a two-way RM-ANOVA design. Assuming a medium effect size (f = 0.25), alpha level (α) of 0.05, statistical power (1 - β) of 0.80, four within-subject conditions (2 layers × 2 sides), and sphericity correction ε = 1.0, the minimum required sample size was calculated to be 52 participants. Therefore, the enrollment of 55 participants was deemed sufficient to ensure adequate statistical power.

## Results

The baseline demographic and clinical characteristics of the participants are summarized in Table 1.

**Table 1:** Participant characteristics (n = 55) n: number of participants; SD: standard deviation; BMI: body mass index; front: front court (net) player; back: back court (baseline) player

Variable	Mean ± SD or n (%)
Age (years)	16.3 ± 0.7
Height (cm)	170.0 ± 6.4
Weight (kg)	58.4 ± 6.6
BMI (kg/m²)	20.2 ± 1.7
Years of playing experience	5.9 ± 2.0
Training frequency (days/week)	6.3 ± 0.5
Total training time (hours/week)	27.4 ± 1.9
Playing position (front/back)	24/31 (43.6%/56.4%)

A two-way RM-ANOVA was conducted to assess the main and interaction effects of layer (superficial vs. deep) and side (dominant vs. non-dominant) on EI (Table 2). The analysis revealed a significant main effect of layer (F(1, 54) = 120.640, p < 0.001, partial η² = 0.691), indicating that EI values were significantly higher in the deep layer compared to the superficial layer. Based on established benchmarks [20], this effect size (partial η² = 0.691) was considered large. In contrast, neither the main effect of side (F(1, 54) = 0.588, p = 0.447, partial η² = 0.011) nor the layer × side interaction (F(1, 54) = 1.322, p = 0.255, partial η² = 0.024) reached statistical significance. Given the non-significant interaction, no post hoc tests for simple main effects were performed.

**Table 2:** Two-way ANOVA results for echo intensity (EI) based on side and layer factors Num: numerator; Den: denominator; ANOVA: analysis of variance

Factor	F-value	Degrees of freedom (Num, Den)	p-value	Partial η²
Side	0.588	(1, 54)	0.447	0.011
Layer	120.640	(1, 54)	<0.001	0.691
Side × layer	1.322	(1, 54)	0.255	0.024

Descriptive statistics (Table 3) further corroborated the main effect of layer. On the non-dominant side, the mean EI in the deep layer was 115.69 (95% CI: 110.38-120.99), compared to 89.84 (95% CI: 87.09-92.58) in the superficial layer. Similarly, on the dominant side, the deep layer showed a mean EI of 113.68 (95% CI: 108.83-118.53), while the superficial layer exhibited a mean EI of 89.59 (95% CI: 86.48-92.71). In both cases, EI values were consistently higher in the deep layer, reinforcing the observed main effect.

**Table 3:** Mean, standard deviation (SD), and 95% confidence intervals (CIs) of echo intensity (EI) by layer and side

Side	Layer	EI mean ± SD	95% CI
Non-dominant	Superficial	89.84 ± 10.25	87.09–92.58
	Deep	115.69 ± 19.60	110.38–120.99
Dominant	Superficial	89.59 ± 11.65	86.48–92.71
	Deep	113.68 ± 21.47	108.83–118.53

## Discussion

This study is the first foundational investigation to non-invasively and quantitatively assess degenerative trends in the superficial and deep layers of the MF using EI as an index, targeting elite-level male high school soft tennis players. A two-way RM-ANOVA conducted on 55 participants revealed a significant main effect for the “layer” factor (superficial vs. deep), indicating that EI in the deep layer was consistently higher than in the superficial layer. In contrast, no significant main effect or interaction involving the “side” factor (dominant vs. non-dominant) was found, suggesting that this structural characteristic was symmetrically present on both sides.

EI serves as a structural parameter reflecting the extent of intramuscular fat infiltration and fibrotic connective tissue and has been reported to show strong correlations with fat fraction measurements derived from MRI and with histological findings from muscle biopsies [17,18]. The observed elevation in EI within the deep MF in this study may imply that degenerative changes-such as fibrosis and fatty infiltration-predominate in the deep layer relative to the superficial layer.

Anatomically, the MF exhibits distinct functional roles by layer. The superficial fibers span multiple vertebral segments and are primarily involved in generating high-torque movements, whereas the deep fibers are situated near the vertebral arch and facet joints and are responsible for maintaining segmental spinal stability [12]. Moreover, the deep layer is predominantly composed of type I muscle fibers and is adapted for sustained postural control [15]; however, due to the characteristics of these fiber types, it may have limited structural adaptability to repetitive and transient high-load stimuli.

Soft tennis is a sport characterized by the repetitive execution of high-intensity, high-velocity trunk movements, such as strokes and serves. These movement patterns inherently place cumulative mechanical stress on the lumbar segments. Under such conditions, the deep MF-responsible for fine segmental control-may be particularly susceptible to chronic microtrauma. This could lead to a more pronounced manifestation of degenerative changes in the deep layer compared to the superficial layer.

Another notable finding of this study is the absence of a significant main effect of side (dominant vs. non-dominant), indicating that the observed degenerative pattern between layers was consistent across both sides. Although soft tennis is a sport characterized by asymmetrical movements, there are numerous phases within trunk motion-such as rotation, lateral bending, and flexion/extension-where bilateral activation of the MF muscles likely occurs in a synergistic manner. Consequently, the cumulative mechanical load imposed on the deep MF may be distributed symmetrically between sides. Moreover, in highly skilled athletes such as those included in the present study, it is conceivable that adaptive mechanisms or compensatory strategies developed through long-term repetitive training may contribute to a more balanced and symmetrical utilization of trunk musculature, thereby minimizing side-to-side differences.

A particularly noteworthy aspect of this study is that all participants were adolescent athletes in their late teens, an age group in which overt signs of muscle degeneration are typically rare. Therefore, the observed differences in EI should be interpreted not as evidence of absolute muscle degeneration but rather as indicative of a relative disparity between the superficial and deep layers. This suggests that the mechanical load characteristics and functional demands may differ between layers, with the deep layer-owing to its role in maintaining segmental spinal stability-being more susceptible to sustained and localized stress accumulation [15]. Such disparities in mechanical loading may lead to an imbalance between layers, manifesting as preferential structural changes in the deep MF.

Furthermore, previous studies on MF muscle degeneration have primarily focused on general populations with chronic LBP and without a history of athletic participation. Some of these reports have indicated that degenerative changes tend to be more pronounced in the superficial layer than in the deep layer [21]. Such findings may reflect disuse-related atrophy or inhibition of superficial muscle activity secondary to pain. In contrast, the present study recruited elite-level male high school soft tennis players, irrespective of the presence or absence of symptoms. Therefore, the observed structural alterations are more likely attributable to overuse rather than disuse. From this perspective, the present findings may represent a distinct pattern of muscle degeneration that is specific to young athletes engaged in rotational sports. This divergence from conventional findings underscores the need for careful interpretation of the results in light of the participants’ athletic background and level of training.

Although this study did not stratify participants based on the presence or absence of LBP and was not designed to directly examine its association, the quantitatively observed higher EI in the deep layer of the MF suggests a potential structural vulnerability relative to the superficial layer. Such interlayer imbalance may reflect a disruption in segmental motor control-namely, dynamic lumbar instability-which could serve as an underlying biomechanical risk factor for future low back disorders. Future research should longitudinally investigate the causal relationship between interlayer differences in EI and the onset of LBP. Such studies would help clarify whether EI asymmetry between the superficial and deep layers of the MF could serve as a clinically useful early screening tool for LBP risk.

Several limitations of the present study should be acknowledged. First, the study population consisted exclusively of elite-level male high school athletes, which limits the generalizability of the findings to female athletes, other age groups, or athletes of different competitive levels. Second, participants were recruited regardless of their current or past history of LBP, and the study design was cross-sectional rather than longitudinal. Therefore, it is not possible to infer any causal link between deep MF degeneration and the onset or persistence of LBP. Third, although EI is a structural parameter reflecting muscle composition, the extent to which it correlates with functional properties such as muscle activity or strength remains unclear. Future studies should incorporate functional assessments, such as electromyographic analysis or isometric strength testing, to establish a more comprehensive understanding of MF degeneration. Fourth, this study assessed EI only at the L4 vertebral level, which may not represent the structural condition of the entire MF muscle or other lumbar segments. Fifth, potential confounding factors such as training volume, playing position (back or front player), and recent injury history were not controlled for and may have influenced MF structure. Sixth, although intra-rater reliability of ROI tracing was confirmed, inter-rater reliability was not tested, and evaluator-dependent bias cannot be ruled out.

Nevertheless, this study offers novel insights by quantitatively demonstrating the possibility of deep layer-dominant muscle degeneration in the MF of young elite athletes, using EI as a non-invasive structural indicator focused on interlayer differences. Previous investigations have primarily targeted middle-aged or elderly individuals or patients with chronic LBP; thus, the observation of such degenerative tendencies in adolescent athletes represents a new contribution. This finding may reflect the localized accumulation of mechanical stress associated with sport-specific demands. As such, it holds both academic and clinical significance in contributing to the foundational understanding necessary for developing preventive strategies against the future onset of LBP in young athletic populations.

## Conclusions

This study quantitatively demonstrated that the deep layer of the MF consistently exhibits higher EI compared to the superficial layer in elite male high school soft tennis players. These findings provide preliminary evidence of a layer-specific tendency toward structural alterations, which may be influenced by the mechanical demands of the sport.

While these results cannot establish a causal link with LBP due to the cross-sectional design, they raise the hypothesis that interlayer disparities in EI could represent an early indicator of spinal vulnerability. Future longitudinal studies are needed to test this hypothesis and clarify whether such disparities are predictive of LBP development in this population.
